# An Analytical and Experimental Study on Cutting Characteristics and Transient Cutting Force Modeling in Feed Directional Ultrasonic Vibration-Assisted Cutting of High Strength Alloys

**DOI:** 10.3390/ma15207388

**Published:** 2022-10-21

**Authors:** Xuelin Chen, Jinyuan Tang, Wen Shao, Bo Hu, Jinxiang Ye

**Affiliations:** 1 State Key Laboratory of High Performance Complex Manufacturing, College of Mechanical and Electrical Engineering, Central South University, Changsha 410083, China; 2 School of Engineering, University of Edinburgh, Edinburgh EH9 3JW, UK; 3 AECC Zhongchuan Transmission Machinery Co., Ltd., Changsha 410083, China

**Keywords:** ultrasonic vibration-assisted cutting, cutting mechanism, cutting force modeling, thermomechanical behaviors

## Abstract

Ultrasonic vibration-assisted cutting (UVC) is progressively being used in machining as it can significantly promote the fabrication process. However, the ultrasonic vibration affecting the cutting process is still controversial. The full-transient cutting process is proposed in this study to analyze the affecting mechanism induced by ultrasonic vibration in the cutting process. This novel model is the first developed based on the fact that ultrasonic vibration would change mechanical behaviors and the cutting process. For example, the reduction of shear flowing stress in the primary shear zone and alteration of the shear angle in the UVC process. Then, considering those coupled effects, a novel model is proposed to determine the average and transient cutting forces. Here, insight and understanding into the physical phenomenon in UVC are provided. The effectiveness of the proposed model is verified by comparison with experimental results and analytical models available in the literature, with cutting parameters varying from macro to micro-scale. The results show that the ultrasonic vibration affects the cutting process in a complicated way, which is determined by transient characteristics, acoustic softening, thermal softening, plowing, and friction. Those effects on cutting performances in the UVC process under various cutting scenarios are investigated and discussed systematically. The average deviation of cutting forces between experiments and values predicted by the proposed model for Ti6Al4V, AISI 1045, and Al6063 is about 7%, 10.2%, and 11%, respectively. The deviation decreases with the increase of cutting speed in the machining of Ti6Al4V, which is different from the machining of other materials. This is contributed by the varied effect of ultrasonic vibration on the cutting process.

## 1. Introduction

The superior properties of high-strength metallic materials such as titanium alloys, nickel-based super-alloys, and high strength steel, etc., as well as their unique performance in a wide range of applications, including aerospace, aviation, automobile, and many other industries, have driven tremendous scientific interest [1]. However, poor machinability and high machining cost have hindered these materials’ usage due to their properties of high strength, low thermal conductivity, and unique chip formation [2]. Machining with traditional methods would inevitably result in large cutting force [3], high cutting temperature, rapid tool wear, and poor surface integrity [4]. Ultrasonic vibration-assisted cutting has emerged as a promising approach to machining various difficult-to-cut materials [5]. The cutting force is a crucial parameter and close related to chip formation, thus can be used as an indicator to predict the cutting performances [6]. Ultrasonic vibration-assisted cutting can reduce the cutting force and its fluctuations and promote the cutting performance to a large extent. The reduction of cutting force was reported to be caused by intermittent cutting [7]. At the same time, the intermittent cutting has also been deemed to contribute to cutting force reduction in radial high-speed ultrasonic vibration cutting [8]. Moreover, it was reported that low-frequency vibration-assisted machining could improve the machinability of difficult-to-cut materials in which intermittent cutting may not occur [9]. On the other hand, the ultrasonic vibration associated with alternation of workpiece materials properties, friction between chip and rake face, and cutting deformation was also reported to contribute to the reduced cutting force [10]. It was found that work-hardening and flow stress drops (also referred to as acoustic softening) existed in ultrasonic vibration-assisted machining [11]. The softening was caused by friction effect, thermal softening, and stress superposition [12]. Although ultrasonic vibration exerted a positive function on the cutting process, Kumabe et al. [13] observed a speed limitation in the practical UVC process. Several types of research found the temperature rises decreased in metal cutting assisted with ultrasonic vibration [14]. At the same time, Doan et al. [15] observed even higher temperatures in elliptical vibration cutting of high-entropy alloy. Chen et al. [16] found that the temperature in the primary shear zone was never steady and fluctuated in the chip segmentation process, which would result in the more complicated deformation and transient characteristics in metal cutting. Patil et al. [17] found that the formation of the shear band and cutting force was reduced in the machining of Ti6Al4V with the presence of ultrasonic vibration. Arefin et al. [18] concluded that, compared to conventional cutting, the intermittent cutting with elastic recovery and strain hardening increased shear angle, lowered chip thickness, and tool-chip contact length in UVC. They claimed the smaller tool-chip contact length was caused by strain hardening, which contributed to more minor secondary deformation. Lotfi et al. [19] argued that tool-chip friction decreased based on the cutting force reduction in 3-D elliptical UV turning of Ti6Al4V. The above literature indicated that the shear flowing stress, the formation process, and the deformation in the primary shear zone and second cutting zone changed significantly in ultrasonic vibration. An in-depth understanding of cutting force reduction would help promote the application of ultrasonic vibration and the break of speed limits.

Considerable research has been devoted to understanding the physical phenomenon of cutting force reduction in the presence of ultrasonic vibration over the years. Zarchi et al. [20] presented an analytical cutting force model for UVC based on the variation of un-deformed chip thickness caused by ultrasonic vibration. The model was verified by the experimental results in UVC of stainless steel. They claimed that the change of un-deformed thickness was attributed to the reduction of cutting force. Paktinat and Amini [21] conducted a numerical and experimental study on ultrasonic vibration drilling of AISI 1045. They found observed the decline of cutting force as high as 45%. Kurniawan et al. [22] observed the reduction of cutting force, higher of stress concentration, and peak cutting tool temperature in ultrasonic elliptical vibration cutting of AISI 1045. Lotfi and Amini [23] found the reduction of cutting forces in the drilling of AISI 1045 when the ultrasonic vibration was added in minimum quantity lubrication. Later, they [24] found lower temperature on tools in their developed finite element model, which is based on the vibratory movement of cutting tool added in conventional drilling of AISI 1045. Elhami et al. [25] proposed an analytical cutting force model in ultrasonic-assisted milling by considering the instantaneous chip thickness and thermal softening. They concluded that the cutting force reduction resulted from the thermal softening in UV milling of hardened AISI 4140. Shen et al. [26] performed pulse-like cutting tests and found that ultrasonic vibration would improve the milling operation through the analysis of the cutting force, surface roughness, and chip morphology in the milling of aluminum alloy. At the same time, the influence of transient cutting parameters caused by ultrasonic vibration is neglected in their study. The ultrasonic vibration cutting force models were also developed based on the LuGre dynamic friction model [27] and the thin shear plane model [28]. Nath et al. [29] pointed out that the instantaneous cutting thickness increased non-linearly from zero and hardly reached the given nominal uncut chip thickness, although the transient velocity of the cutting tool changes continuously. Chou [30] concluded that the ultrasonic vibration wave decreased cutting forces by reducing the seizure friction and accelerating the chip removal when the ultrasonic vibration wave was applied to the tool-chip interface. Jamshidi and Nategh [31] found that the cutting force reduction could be attributed to a higher decrease in normal force than that of the frictional force, rather than the lower friction on the tool-chip interface. However, the effects of transient tool velocity on transient shear angle and transient cutting depth were neglected in those models.

With regards to this issue, several attempts have been made towards modeling the modeling of cutting force based on the chip area, contact length, and materials properties in UVC. Nategh et al. [32] developed a kinematics model for elaborating the machining interruption and restart in UVC. Then, the cutting force model was proposed based on the dynamic analysis of UVC [33]. It was found that the inertial effect of chip acceleration affected the shear angle and peak cutting force. They claimed that the ultrasonic vibration affected the cutting process in UVC by chip acceleration. Later, they further pointed out that the reduction of cutting force was contributed by the increased burnishing effect caused by tool motion [34]. Prabhakar et al. [35] modeled the acoustic softening effect based on the extended dislocation density-constitutive model. They claimed that the mechanical properties of the workpiece in the contact area were altered with the superposition of UV, and the residual hardening was incorporated in their model. Zhang et al. [36] proposed the analytical force model based on geometric modeling and Shaffer’s slip-line solution considering the transient thickness of cut, transient shear angle, and transition characteristic of friction reversal. The shear angle in their model was constantly based on the measured chip thickness, fixed uncut chip thickness, and rake angle. Lin et al. [37] presented a cutting force model for oblique elliptical vibration cutting in which transient cutting thickness and transient shear angle were also considered. The shear-flowing stress was set as constant in the cutting process. It was controversial as the shear flowing stress was affected by strain and strain rate, which were determined by cutting velocity and acoustic vibration cutting speed. Bai et al. [38] proposed a cutting force model with consideration of transient cutting thickness and transient shear angle was further presented. Since the three stages were assumed to be divided into the whole ultrasonic cutting process, which is different from the fact that the shear angle was changing in the whole cutting process. And the transient cutting force was not fully modeled. Verma et al. [39] proposed the cutting force model considering the acoustic softening, intermitted cutting, and helix angle effect. While the modeling of shear angle was based on the constant of cutting velocity and uncut chip thickness. Transient characteristics caused by ultrasonic vibration were neglected. Zhang et al. [40] conducted high-speed ultrasonic vibration-assisted cutting experiments to study the separation effect undercutting condition of the worn tool and transient clearance angle. They concluded that the reduction of cutting force resulted from the separation effect and would be improved by optimizing the combination of feed rate, phase shift angle, and negative clearance angle. Han and Zhang [41] confirmed that the separating characteristic contributed to radial cutting force reduction by the experimental results in UV-assisted milling of Ti6Al4V. Liu et al. [42] developed an analytical cutting force model in UV-assisted milling. They claimed that the acceleration of the chip induced by ultrasonic vibration was the main cause of cutting force reduction in UV-assisted milling. Verma and Pandy [43] have also confirmed that the average cutting forces were reduced by intermittent cutting with a statistical model in which the influence of process parameters on cutting force was evaluated. Gupta et al. [44] studied Tribological performance based on machinability in the cryogenic cutting of Ti6Al4V. Later, they [45] studied the tool wear pattern in the machining of titanium alloy with the assistant of hybrid cooling. Agrawal et al. [46] found the reduction of active energy and increase in energy efficiency in the presence of hybrid turning techniques. Fardnam et al. [47] observed the obvious improvement of surface roughness when ultrasonic intensity increased from 15 W/cm^2^ to 22.5 W/cm^2^ in face milling of AISI 1045 and 7075 aluminum. Feng et al. [48] developed a physics-based analytical temperature model for ultrasonic vibration-assisted cutting. They observed the decrease in temperature when the ultrasonic vibration amplitude increased in the machining of AL 6063. Airao et al. [49] found the ultrasonic vibration reduced surface roughness and power consumption compared to conventional turning of Inconel 718. Airao and Nirala [50] observed the increased shear angle and decreased tool-workpiece contact ratio when ultrasonic vibration amplitude and frequency increased in the machining of SS 304. Meanwhile, Airao et al. [51] applied ultrasonic vibration with minimum quantity lubrication and liquid carbon dioxide in the machining of Ti6Al4V and found a significant reduction of specific cutting energy. In an overview of the mentioned research, the transient cutting force in UVC was not completely modeled, and a fully transient cutting force model would promote an in-depth understanding of the reduced cutting force and cutting energy.

In the present research, the full-transient cutting process is first modeled to investigate the ultrasonic vibration affecting the mechanism of cutting process, and the model is developed based on the fact that ultrasonic vibration would change mechanical behaviors, such as reduction of shear flowing stress in the primary shear zone and alter of the shear angle in UVC process. Then, the time-varying and average cutting force models are presented by the metal cutting mechanism. The acoustic softening coefficient is determined based on the experimental results. The proposed model is further compared with experimental results in machining Ti6Al4V, AISI1045, and Al6063 samples, and several analytical cutting force models are available in the literature. Finally, an in-depth analysis of the cutting force reduction phenomenon and ultrasonic vibration affect mechanism on the cutting process in UVC are presented.

## 2. Theoretical Model

### 2.1. Modeling of Cutting Process

According to the mechanical kinematic characteristics of turning and orthogonal cutting processes [6], the feed rate and its turning direction correspond to uncut chip thickness (also called undeformed chip thickness). The changes in feed rate induced by ultrasonic vibration are equal to that of uncut chip thickness, which contributes to the alternations of strain and strain rate in the primary shear zone. The trajectory of the cutting tool also changes, which alters the strain and shear angle. Several models have been proposed to model the primary shear zone in the chip segmentation process, such as the thin shear plane model [29], two cutting areas with four boundaries of cutting speed [52], non-equidistant primary shear zone [53], slip-line field modeling [54], parallel shear zone [55]. It should be noticed that the single shear model is the most widely used model, which is the benchmark of the chip above segmentation model. The shear model could also model the primary shear zone in ultrasonic vibration-assisted cutting [37,38,40]. Therefore, adopted to analyze the primary site in this study. The coupling effect of cutting parameters, cutting process, strain, stress, temperature, and acoustic effect induced by ultrasonic vibration would emerge when the ultrasonic vibration is given in the feed direction. The ultrasonic vibration is thus imposed in the feed direction to investigate that coupling effect in metal cutting.

**Figure 1:**
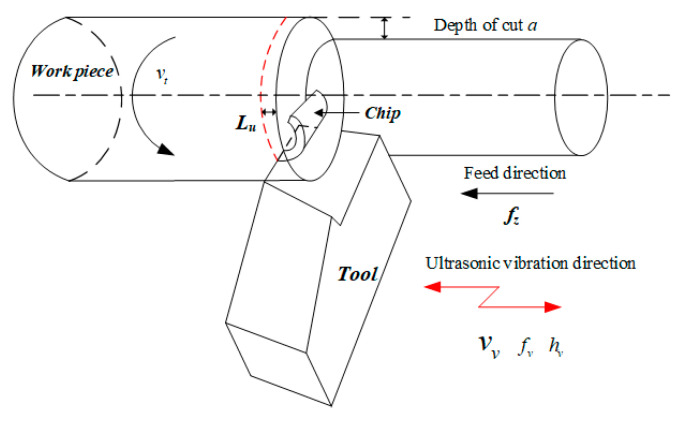
Illustration of the acoustic vibration-assisted turning.

**Figure 2:**
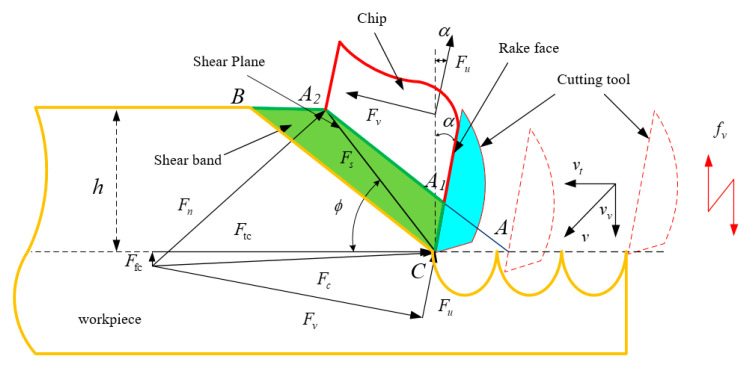
Principles of acoustic vibration-assisted cutting.

This study’s vibration direction in the ultrasonic vibration-assisted turning process is the feed direction. The schematic diagram of the cutting process and the corresponding principle are presented in Figure 1 and Figure 2. The feed rate in UVC with a vibration frequency of *f_z_* can be developed as

(1) $$f_{z}=h+h_{v}\sin(2\pi f_{v}t+\theta )$$

where, $h_{v}$ is the vibration amplitude in the feed direction, $f_{v}$ is tool vibration frequency, θ is the initial phase shift angle when machining is beginning, it is set as 0 in most cases in this study, *t* is the time, *h* is the feed rate without acoustic vibration.

Based on the diagram of turning transfer to orthogonal cutting [56], the un-deformed chip thickness *h_z_* in orthogonal cutting is equal to feed rate *f_z_* in ultrasonic vibration-assisted turning.

(2) $$h_{z}=f_{z}=h+h_{v}\sin(2\pi f_{v}t+\theta )$$

In the stable cutting stage, the cutting velocity *v_t_* is equivalent to the line speed of the cutting edge, which is expressed as

(3) $$v_{t}=\pi nD_{r}$$

where *D_r_* and *n* are the diameters of workpiece and rotation speed.

The acoustic vibration speed is the synthetic cutting speed, which is determined by the moving speed of the cutting tool induced by ultrasonic vibration and cutting speed. The acoustic vibration speed *v* is determined by the following equation

(4) $$v=\sqrt{v_{t}{}^{2}+v_{v}{}^{2}}$$

The acoustic vibration speed of cutting tool in the vibration direction *v_v_* can be developed as the time-derivative of tool position.

(5) $$v_{v}=2\pi f_{v}h_{v}\cos(2\pi f_{v}t+\theta )$$

**Figure 3:**
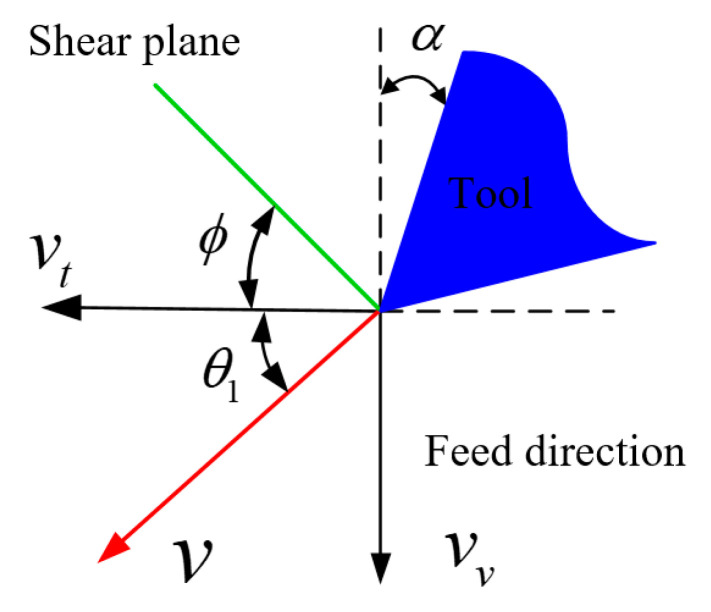
Diagram of cutting speed in orthogonal UVC.

Figure 3 shows the relationship between convectional cutting speed, acoustic vibration speed, and cutting speed in acoustic vibration-assisted turning and orthogonal cutting mechanism.

The angle between the traditional cutting velocity and resultant cutting velocity *θ*_1_ is developed as

(6) $$\theta_{1}=\arctan(\frac{v_{v}}{v_{t}})$$

The effective angle of rake face in acoustic vibration cutting is presented as

(7) $$\alpha_{v}=\alpha +\theta_{1}$$

where α is the rake angle of the cutting tool.

The shear angle in acoustic vibration-assisted cutting is obtained as

(8) $$\phi_{v}=\phi +\theta_{1}$$

where ϕ is the shear angle without acoustic vibration.

Experimental studies proved that the shear angle was related to the cutting parameters and material properties [57]. The analytical shear angle is expressed as

(9) $$\phi =\frac{5}{8}\alpha +\frac{1}{2}\cos^{-1}\left[\exp\left(-52.5\times 10^{-3}{\left(\frac{\tau }{100c\rho }\right)}^{\xi_{1}}{\left(\frac{vh\times 10^{-3}}{60\omega }\right)}^{\xi_{2}}\right)\right]$$

where α is the rake angle, $\tau_{s}$ is the yield strength, *c* is the specific heat, ρ is the density of work-piece, ω is the thermal conductivity of the work-piece, $\xi_{1}$ and $\xi_{2}$ are the constants that control the average temperature of chip contributed by pure friction, which are determined by thermal-mechanical properties of the material. The constants are defined as 0.8 and 0.4 according to the temperature developed in [58]. It was claimed that the model was also applicable for carbon, alloyed, steel, and stainless based on thermos-dynamic analysis and experimental data. Toropov and Ko [59] proved the model by machining stainless steel and aluminum alloy. Verma et al. [39] showed that Equation (9) could predict shear angle effectively in UVC of aluminum alloy, and the coefficient depended on the thermomechanical properties. Table 1 shows the thermomechanical properties of aluminum, titanium and stainless steel. The thermomechanical properties of titanium alloy fall between those of aluminum alloy and stainless steel. Thus, the model is also applicable for titanium alloy.

In ultrasonic vibration-assisted cutting, the dynamic load applied in cutting area by the cutting tool affects the stress of cutting area by changing the strain, and strain rate with the presence of ultrasonic vibration. Johnson-Cook (J-C) model is used model the stress with input of strain, and strain rate incorporates the changes induced by ultrasonic vibration to a certain extent.

The material yield stress in the shear area which is close related with the temperature, strain and strain rate which changes with the resultant speed [62]. The standard J-C model is used to evaluate the material yield stress.

(10) $$\sigma =\left(A+B\varepsilon_{s}{}^{n}\right)\left\{1+m\ln\frac{{\dot{\varepsilon }}_{s}}{{\dot{\varepsilon }}_{0}}\right\}\left\{1-{\left(\frac{T_{v}-T_{r}}{T_{m}-T_{r}}\right)}^{c_{1}}\right\}$$

where $\varepsilon_{s}$, ${\dot{\varepsilon }}_{s}$,${\dot{\varepsilon }}_{0}$ are strain, strain rate, reference strain rate of shear band in acoustic vibration-assisted cutting. $T_{v}$, $T_{m}$, $T_{r}$ are temperature of shear area in acoustic vibration-assisted cutting, melting temperature and room temperature. *A*, *B*, *C*_1_, *m* and *n* are material constants in Johnson-Cook model.

Based on the shear strain energy yield criterion, the equations for uniaxial flowing stress relating to the plane strain shear flowing stress are developed as follows

(11) $$\tau =\frac{1}{\sqrt{3}}\sigma$$

(12) $$\varepsilon_{v}=\sqrt{3}\varepsilon_{s}$$

(13) $$\varepsilon_{v}=\sqrt{3}{\dot{\varepsilon }}_{s}$$

Then, the plane shear flowing stress can be developed as

(14) $$\tau =\frac{1}{\sqrt{3}}\left(A+B{\left(\frac{\varepsilon_{v}}{\sqrt{3}}\right)}^{n}\right)\left\{1+m\ln\frac{{\dot{\varepsilon }}_{v}}{{\dot{\varepsilon }}_{0}}\right\}\left\{1-{\left(\frac{T_{v}-T_{r}}{T_{m}-T_{r}}\right)}^{c_{1}}\right\}$$

A number of studies [11,12] showed that the ultrasonic vibration lowered the material strength by the activation of blocked dislocations. This effect is regarded as the thermal softening and acoustic softening effect. The ultrasonic vibration has been considered the source of activation energy that causes dislocation annihilation and sub-grain formation. However, it has been proved that thermal softening is caused by the high intensity of heat source in the machining of low thermal conductivity materials. This can be achieved by the high velocity of the cutting tool, which is synthesized and controlled by cutting and ultrasonic vibration velocities since one objective of this study is to comparatively analyze the effect of ultrasonic vibration on stress induced by changes in cutting parameters with published theoretical models. The stress lowering effect caused by the activation of blocked dislocations already considered in those existing theoretical models, is neglected in the proposed model.

The models of strain and strain rate in the primary shear zone without ultrasonic vibration have been proposed in several studies [56,60]. The strain and the strain rate of the primary shear area in ultrasonic vibration-assisted cutting can be developed as

(15) $$\varepsilon_{v}=\frac{\cos\alpha_{v}}{2\sin\phi_{v}\cos\left(\phi_{v}-\alpha_{v}\right)}$$

(16) $${\dot{\varepsilon }}_{v}=\frac{\nu \cos\alpha_{v}}{\Delta d\cos(\phi_{v}-\alpha_{v})}$$

where Δd is the thickness of the shear zone.

Based on the empirical equations proposed in Ref. [39], the empirical relation for the shear zone is presented as

(17) $$\Delta d=\frac{h_{z}}{5.9\sin\phi_{v}}$$

Based on the temperature model of shear band in metal cutting [39,60], averaged temperature of the shear band for AL6063, steel, Ti6Al4V are given as follows, respectively.

(18) $$T_{v}=T_{r}+\eta \psi (1-\lambda_{s})\left(\frac{\tau \cos\alpha_{v}}{\rho c\cos\left(\phi -\alpha_{v}\right)\sin\phi }\right)$$

(19) $$T_{v}=T_{r}+\lambda_{h}(1-\lambda_{s})\left(\frac{\tau \cos\alpha_{v}}{\rho c\cos\left(\phi -\alpha_{v}\right)\sin\phi }\right)$$

(20) $$T_{v}=T_{r}+\frac{\varpi \tau {\dot{\varepsilon }}_{v}}{\rho cv\sin\phi }$$

where $\lambda_{h}$ is the factor that the plastic work done outside the shear zone, η is a coefficient corresponding to the fraction of the strain energy transformed into heat, ψ is the coefficient for thermal softening coefficient, ϖ is the Taylor-Quinney coefficient, $\lambda_{s}$ is the proportion of the heat conducted into the workpiece, τ is the flow stress of shear band.

According to the empirical equation proposed in Ref. [56], the proportion of heat transferred into the workpiece is given by

(21) $$\lambda_{s}=\{\begin{array}{c} \begin{array}{cc} 0.5-0.35\log\left(R_{t}\tan\phi_{v}\right) & 0.04\leq R_{t}\tan\phi_{v}\leq 10 \end{array}\\ \begin{array}{cc} 0.3-0.15\log\left(R_{t}\tan\phi_{v}\right) & 10<R_{t}\tan\phi_{v} \end{array} \end{array}$$

where $R_{t}$ is a non-dimensional thermal constant.

(22) $$R_{t}=\frac{\rho cvh_{z}}{\omega }$$

where ω is the thermal conductivity of work material.

Based on Equations (15)–(22), The shear flow stress and shear angle in the cutting of Al 6063, steel, Ti6Al4V can be calculated.

The frictional angle can be determined based on the empirical equation of frictional angle in Ref. [56] in metal cutting.

(23) $$\beta_{v}=\tan^{-1}\left(\frac{1}{1-\tan\alpha_{v}}\right)-\phi_{v}$$

### 2.2. Modeling of Cutting Forces

In the acoustic cutting process, the intermittent cutting may happen when the amplitude of acoustic vibration is larger than the feed rate in the reverse direction. The cutting force along the main shear plane in the coordinate system, which is based on the synthetic cutting speed coordinate systems, is estimated by

(24) $$F_{s,v}=\tau \mathrm{sgn}(h)\frac{h_{v}}{\sin\phi_{v}}a$$

(25) $$\mathrm{sgn}(h)=\{\begin{array}{c} \begin{array}{cc} 1 & h_{t}\geq h_{v}\sin(2\pi f_{v}t+\theta ) \end{array}\\ \begin{array}{cc} 0 & h_{t}<h_{v}\sin(2\pi f_{v}t+\theta ) \end{array} \end{array}$$

where *a* is the cutting depth as well as the width of the un-deformed chip.

The resultant force, tangential cutting force, and feed cutting force in the synthetic cutting speed coordinate systems are expressed as.

(26) $$F_{c,v}=\frac{F_{s,v}}{\cos(\beta_{v}-\alpha_{v}+\phi_{v})}$$

(27) $$F_{tc,v}=\cos(\beta_{v}-\alpha_{v})\frac{F_{s,v}}{\cos(\beta_{v}-\alpha_{v}+\phi_{v})}$$

(28) $$F_{fc,v}=\sin(\beta_{v}-\alpha_{v})\frac{F_{s,v}}{\cos(\beta_{v}-\alpha_{v}+\phi_{v})}$$

**Figure 4:**
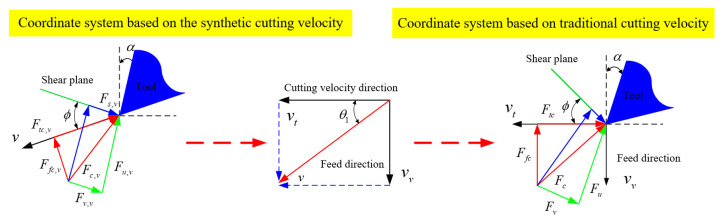
The coordinate relationship between synthetic cutting speed and traditional cutting speed.

**Figure 5:**
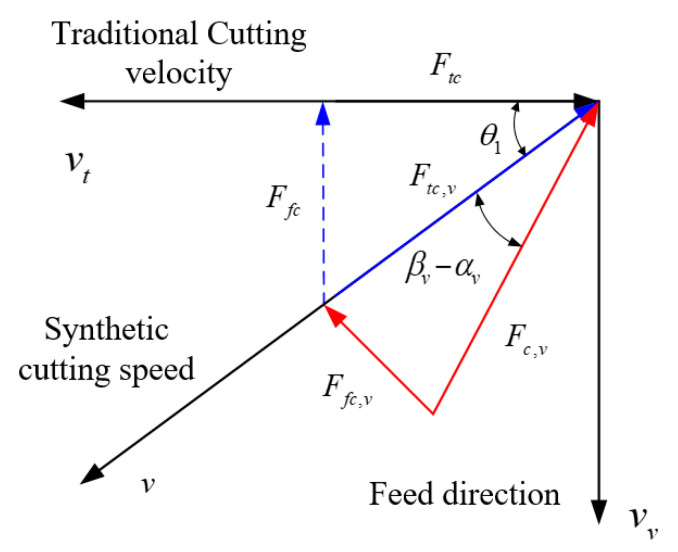
The relationship of cutting force in two coordinate systems.

As the cutting forces are measured based on the coordinate system without ultrasonic vibration; the forces should be normalized to this coordinate system. Figure 4 shows the relationship between coordinates in synthetic cutting speed and cutting speed in traditional machining. The relationship between two coordinate systems can be established by the relationship between the synthetic cutting speed and the cutting velocity in conventional machining. The relationship of feed and tangential cutting forces in the two related coordinate systems in ultrasonic vibration-assisted cutting is presented in Figure 5.

The tangential and feed cutting force in coordinates of traditional cutting speed, which is equal to the measuring coordinates, are expressed as

(29) $$F_{tc}=F_{c,v}\cos(\beta_{v}-\alpha_{v}+\theta_{1})$$

(30) $$F_{fc}=F_{c,v}\sin(\beta_{v}-\alpha_{v}+\theta_{1})$$

The averaged cutting force in one cycle of acoustic vibration is expressed as.

(31) $$F_{tc,ave}=\frac{\int_{0}^{t_{1}}F_{tc}dt}{t_{1}+t_{2}}$$

(32) $$F_{fc,ave}=\frac{\int_{0}^{t_{1}}F_{fc}dt}{t_{1}+t_{2}}$$

where *t*_1_ and *t*_2_ are duration time with and without contact between the cutting tool and workpiece in the cycle.

The period of acoustic vibration is developed as

(33) $$T=\frac{1}{f_{v}}$$

(34) $$T=t_{1}+t_{2}$$

*t*_1_ and *t*_2_ are obtained from the relationship between feed rate and amplitude of acoustic vibration, which are expressed as

(35) $$\begin{array}{l} t_{1}=\{t,h+h_{v}\sin(2\pi f_{v}t+\theta )\geq 0,0\leq t\leq T=\frac{1}{f_{v}}\}\\ t_{2}=\{t,h+h_{v}\sin(2\pi f_{v}t+\theta )<0,0\leq t\leq T=\frac{1}{f_{v}}\} \end{array}$$

**Figure 6:**
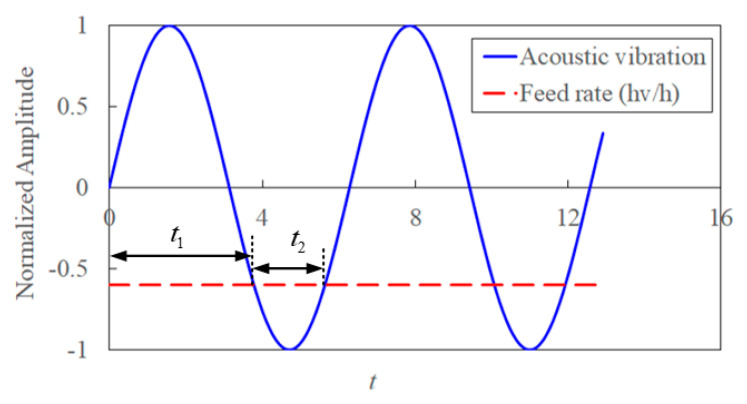
The relationship of cutting in and out in UVA machining.

In one acoustic vibration cycle, the duration time of cutting in and cutting out in Figure 6. The time of cutting out is defined as *t*_2_, which is developed as

(36) ti=arcsin(−hvh)−θ2πfv,i=1,2

According to the developed tool angle conversion and cutting force relationship between orthogonal cutting and oblique cutting in Ref. [56], the cutting forces $F_{x}$, $F_{y}$, $F_{z}$ in x, y, and z directions are calculated. The detailed calculation producers are presented in Appendix A.

### 2.3. Modeling of Acoustic Softening Constant

According to the study on ultrasonic stress [12], acoustic stress is expressed as

(37) σ=ρcU

where ρ is the material density, *U* is the velocity of a specific material point, and *c* is the velocity of sound in the medium.

Ultrasonic intensity is developed as

(38) $$I=\frac{\sigma^{2}}{\rho c}=\rho cU^{2}$$

The ultrasonic angular frequency is given by

(39) ω=2πf

The particle velocity is developed as

(40) U=ωξ=2πfξ

where ξ is the deformational distance, *f* is the ultrasonic vibration frequency.

The stress in the presentence of ultrasonic vibration is formulated as

(41) $$\sigma =\sigma_{{}_{n}}{\left(1-DI\right)}^{e}$$

where $\sigma_{{}_{n}}$ is stress without vibration.

Then, the ultrasonic stress is derived as

(42) $$\sigma =\sigma_{{}_{n}}{\left(1-4\pi^{2}f^{2}\xi^{2}D\rho c\right)}^{e}$$

where the constants *D* and e are the acoustic coefficients.

When the constant e is set as 1, the stress under ultrasonic vibration is expressed as

(43) $$\sigma =\sigma_{{}_{n}}(1-4\pi^{2}f^{2}\xi^{2}D\rho c)$$

The constant *D* is calculated by the stress with and without ultrasonic vibration.

## 3. Experiments

### 3.1. Ultrasonic Vibration Apparatus

**Figure 7:**
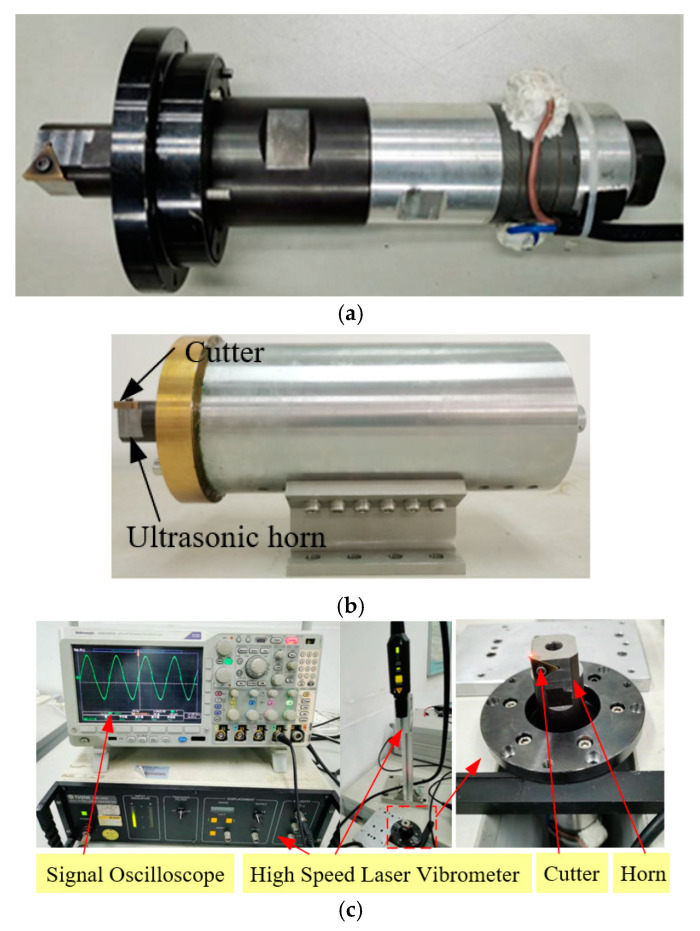
The assembled apparatus (**a**) without protective shell, (**b**) with protective shell, and (**c**) its testing experiments.

**Figure 8:**
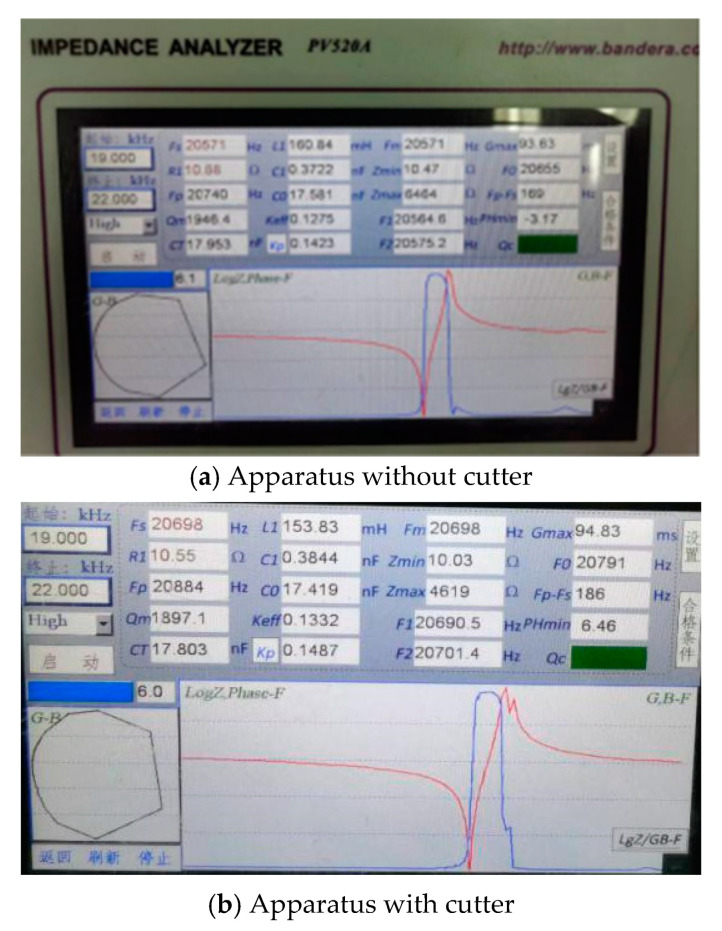
The testing origin data of ultrasonic vibration apparatus.

**Figure 9:**
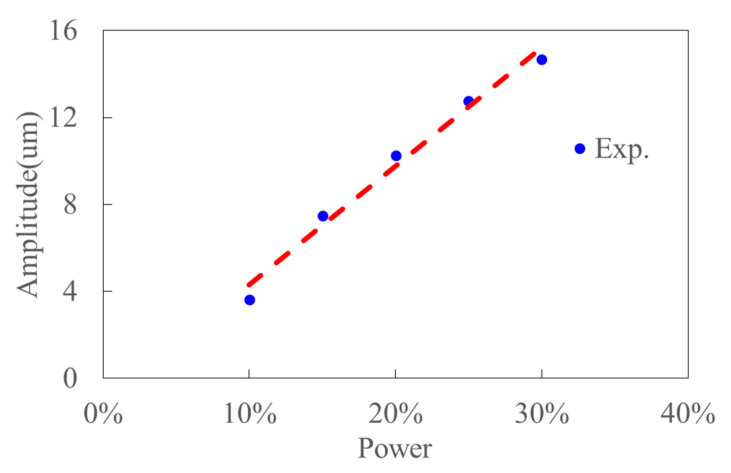
The variation of ultrasonic amplitude with power for ultrasonic vibration apparatus.

Figure 7 shows the ultrasonic vibration apparatus and measurement equipment. The equipment consists of a Signal Oscilloscope (Tektronix DPO4104, TEKTRONIX, INC., Beaverton, OR, USA), an Impedance Analyzer (Bandera PV520A, Bandera electronics Co., Ltd., Beijing, China) and a High Speed Laser Vibrometer (Polytec HSV 2002, Polytec GmbH, Waldbronn, Germany). And the frequency, impedance and amplitude of ultrasonic apparatus can be obtained in the testing experiments. Table 2 shows the results of frequency and impedance, which are obtained by the Impedance Analyzer. The original testing data are presented in Figure 8. This shows the performance of ultrasonic vibration apparatus is stable and reliable. The rated power of the ultrasonic generator (Super Sonic Co., Ltd., Dongguan, Guangdong, China) is 1500W. Figure 9 presents the experimental ultrasonic amplitudes when the ultrasonic generator outputs 10%, 15%, 20%, 25% of the rated power.

### 3.2. The UVC Experiments

**Figure 10:**
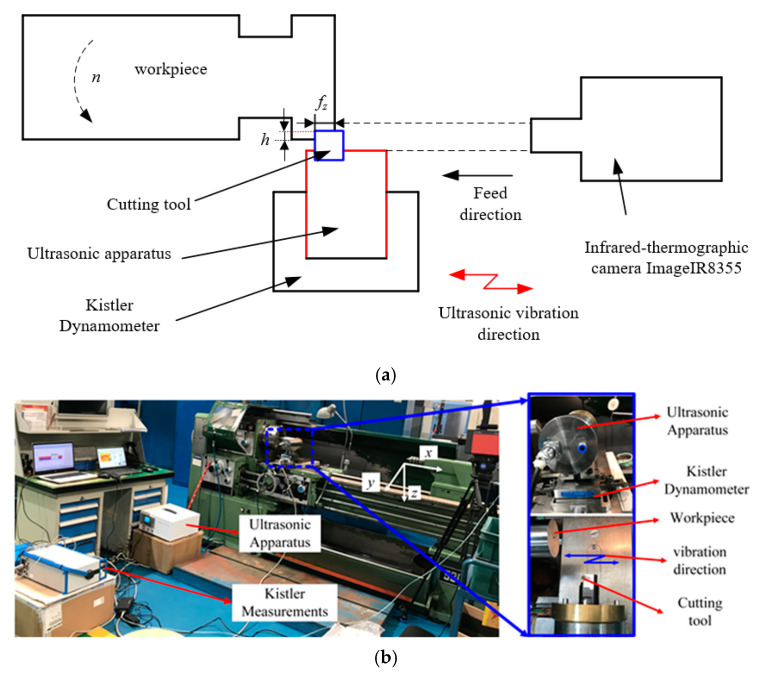
Presentation of the experiment by: (**a**) schematic and (**b**) experimental setup.

The UVC experiments is conducted on a conventional turning machine. Figure 10 shows the experimental set up for UVC. The ultrasonic vibration is imposed in feed direction whose basis has been presented in Section 2.1, as well as the chip transportation would occur if the ultrasonic vibration direction was imposed in the feed direction [30]. The cutting force is measured by Kistler 9257B (Kistler Group, Eulachstrasse, Winterthur, Switzerland). The PVD coated TiAlN with a rake angle of 0° and a cutting edge angle of 60°is employed in the cutting tests. Titanium alloy Ti6Al4V bar with a diameter of 59 mm is used as the workpiece. The resonant frequency is 20283 Hz when this cutter replaces the original cutter. Based on the ultrasonic vibration amplitude and experimental cutting parameters in published Refs. [6,7,8,9,10,11], the cutting parameters for the experiment are planned and presented in Table 3. Each cutting experiment has repeated ten times and the experimental cutting force are determined by averaging those cutting force value.

### 3.3. Presentations of Experiments in Published References

The material properties and Johnson-Cook parameters of aluminum alloy, titanium alloy and AISI steel are obtained from Refs. [39,60,61,63] and presented in Table 1 and Table 4. Room temperature is set as 300K. The composite of Ti6Al4V has been detected by Tescan MIRA3LMU equipment (TESCAN, INC., Brno, Czech). And the composite is presented in Table 5. The experimental cutting force of aluminum alloy in ultrasonic vibration-assisted milling are presented in Ref. [39]. The transient cutting force of AISI 1045 in the ultrasonic vibration cycle was measured in the orthogonal acoustic vibrational assisted cutting experiments [40]. The material chemistry of AISI 1045 and aluminum alloy workpieces are thus not presented.

Table 6 provides the acoustic softening constants and thermal convection coefficients in metal cutting. The influence of ultrasonic vibration on material properties of aluminum alloy are obtained from Ref. [39]. The influence of ultrasonic vibration on AISI steel are calculated based on the stress in ultrasonic vibration cutting experiments [40]. Acoustic softening constant 1 was calculated based on the modeling in Equations (35)–(41) under ultrasonic vibration amplitude of 8 um. The detailed description for determining the acoustic softening constants of AISI 1045 is presented in Appendix B. The acoustic softening constants of Ti6Al4V are not given as the constants are not used in this study.

## 4. Results and Discussion

In this section, the result of Ti6Al4V is discussed and compared with that of the published references first. Then, the calculated transient characteristics of AISI steel and the cutting force of aluminum alloy are further verified and compared with those of the simplified models of the published references [36,37,39]. The friction coefficients in Ref. [37] for AISI steel and aluminum alloy are set as 0.16 and 0.3302.

### 4.1. Machining of Ti6Al4V

**Figure 11:**
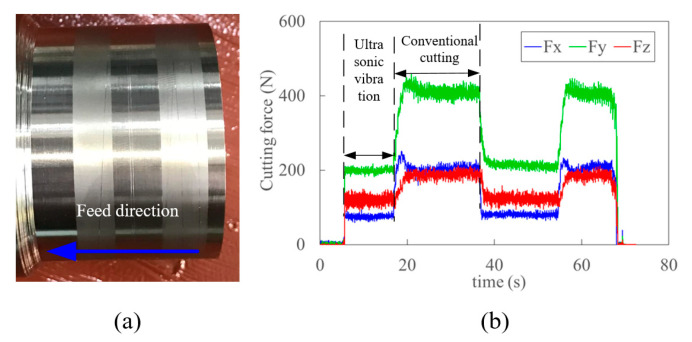
The typical work-piece (**a**) and cutting forces (**b**) in cutting with and without ultrasonic vibration.

**Figure 12:**
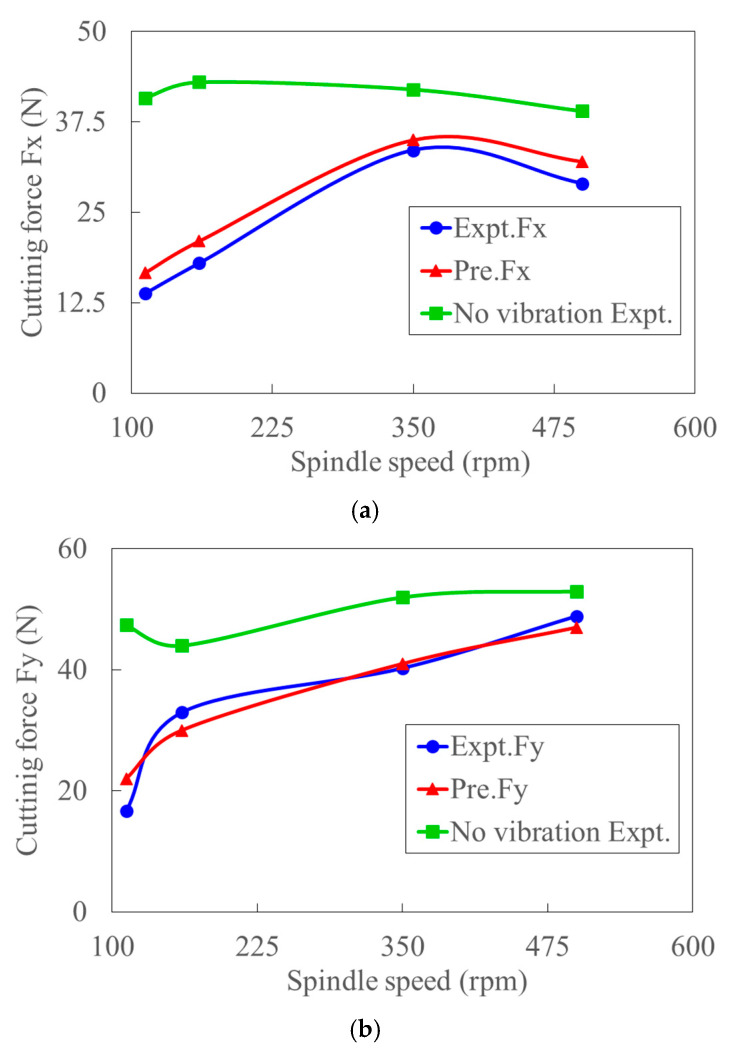

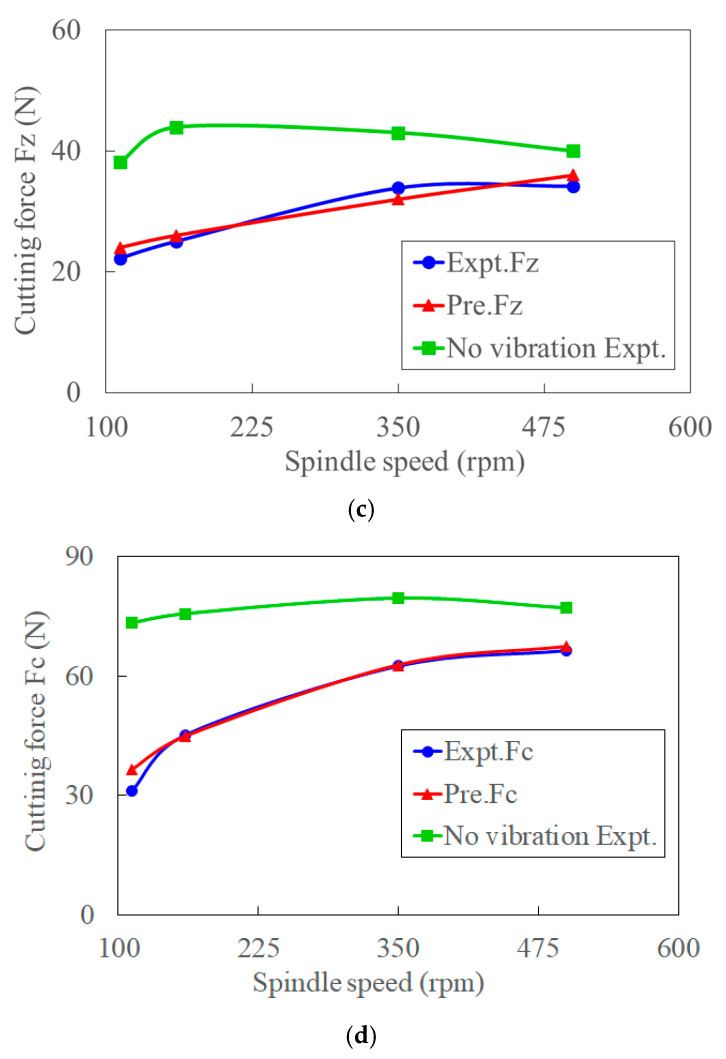
Comparison between the predicted and experimental cutting forces with ultrasonic amplitude of 14.75 um and cutting depth of 0.3 mm: (**a**) Fx, (**b**) Fy, (**c**) Fz, and (**d**) Fc.

Figure 11 shows the typical workpiece and time-varying cutting force with and without ultrasonic vibration. Compared to conventional cutting, the much lower cutting force and smoother finish of the surface were observed in UVC. The cutting forces of Fx, Fy, and Fz in x, y and z directions, as well as the total cutting force Fc, in the oblique cutting condition, are calculated from orthogonal cutting based on the empirical approach in Ref. [56]. A good agreement between the experimental and predicted cutting forces and obvious force reduction in UVC is observed in Figure 12. Based on the critical cutting speed for effective cutting [30], the critical cutting velocity for Ti6Al4V is 112.2 m/min in this study. When the spindle speeds (rpm) are 112, 160, 350, 500, the corresponding cutting velocities (m/min) are 20.75, 29.64, 64.84, 92.63, respectively. With the increase of cutting speed, the reduction rate of cutting force decreases. The reason for this might be the existence of intermitting cutting when the feed rate is lower than the ultrasonic vibration amplitude. The relative larger difference between the predicted and measured cutting forces at low cutting speed is caused by the work-hardening and acoustic softening effects, which are ignored in the proposed model. The difference reduces significantly because the work-hardening effect decreases rapidly with the increased cutting speed [2]. The trend of cutting forces (Fx, Fz) in convectional cutting, which increases first and then decreases with the increase of cutting speed, also indicates this. The thermal softening effect gradually enhances with the increased cutting velocity while the acoustic softening effect weakens. It is worth noting that the feed cutting force Fx decreases significantly when spindle speed reaches 500 rpm. This might also be caused by the intermit cutting process when the feed rate is lower than the ultrasonic amplitude and enhanced thermal softening effect. Therefore, an intrinsic critical velocity could exist in UVC. The deviation decreases rapidly from 17.8% to 2% in total cutting force when cutting speed increases from 20.75 m/min to 92.63 m/min. The average deviation of the total experiments and predictions is about 7%.

### 4.2. Machining of AISI 1045

#### 4.2.1. Shear Angle

**Figure 13:**
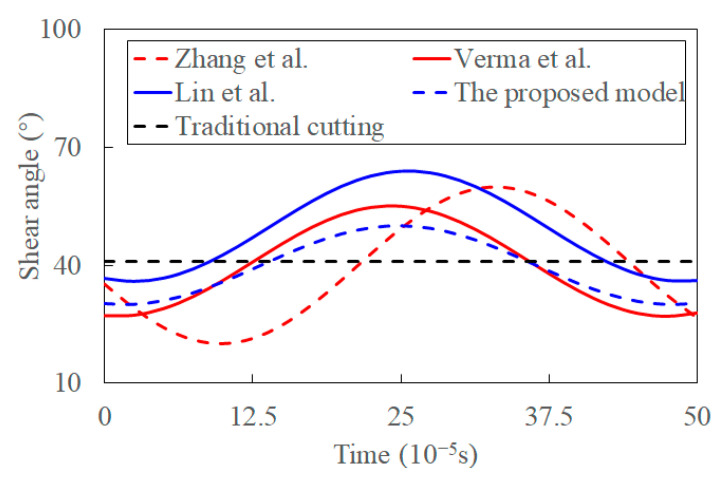
The evolution of shear angle in chip formation process (h = 0.005 mm, hv = 8 um, n = 652 r/min, θ = 36°, *D_r_* = 12 mm).

Figure 13 presents the predicted transient shear angle in ultrasonic vibration and experimental shear angle in traditional cutting. The shear angle predicted by Zhang et al. is different from the other models. The reason might be that the influence of the various shear stress is neglected. The shear stress, which significantly influences the shear angle, is synthetically determined by the temperature and transient cutting factors. The shear angle changes drastically, and its fluctuations are highest when the variance of shear flowing stress induced by ultrasonic vibration is ignored. This is because only the transient tool velocity and rake angle are considered in the model. It seems that the ultrasonic vibration promotes the cutting process by reducing the fluctuation of shear angle. This is consistent with the experimental fact that the decreasing cutting force and its reduced amplitude are in the presence of ultrasonic vibration [34].

The fluctuation of shear angle decreases, and its average value seems to increase in Ref. [37], where the transient tool velocity and its angle are considered. The influence of shear flowing stress on a shear angle is considered by the maximum shear flowing stress principle, where the shear flow stress is set as constant. However, it is controversial that the maximum shear flowing stress is varied in UVC. The influence of shear-flowing stress is significant in determining the shear angle. Verma et al. [39] analytically predicted the transient shear angle based on the analysis of shear flowing stress and empirical shear angle. While the effect of thermal softening, acoustic softening, strain, and temperature alternations induced by ultrasonic vibration on the shear-flowing stress are neglected in their study. Compared to the value predicted by Lin et al. [37], the reduction of fluctuated shear angle induced by the acoustic softening was more significant than that caused by transient cutting parameters and the maximum shear flowing stress principle. Fluctuation of shear angle decreases slightly when the coupling effect of shear flowing stress and shear angle induced by transient cutting parameters is considered in the proposed model. Shear angle in traditional cutting is calculated by empirical equations with the input of cutting parameters and material properties [58,59]. The chip segment is the source of the fluctuating cutting forces in traditional machining [6,65]. The predicted shear angle is an average value as it varies during the cutting process. The predictions are more significant than those in conventional cutting, consistent with the experimental observations [65]. The reason for this might be the elastic recovery and strain hardening [19]. Thus, the influence of transient cutting parameters, acoustic softening, shear flowing stress, and thermal softening on a shear angle is gradually increasing. The ultrasonic vibration decreases the fluctuation of shear angle, and this stabilizes the cutting process.

#### 4.2.2. Shear Flowing Stress

**Figure 14:**
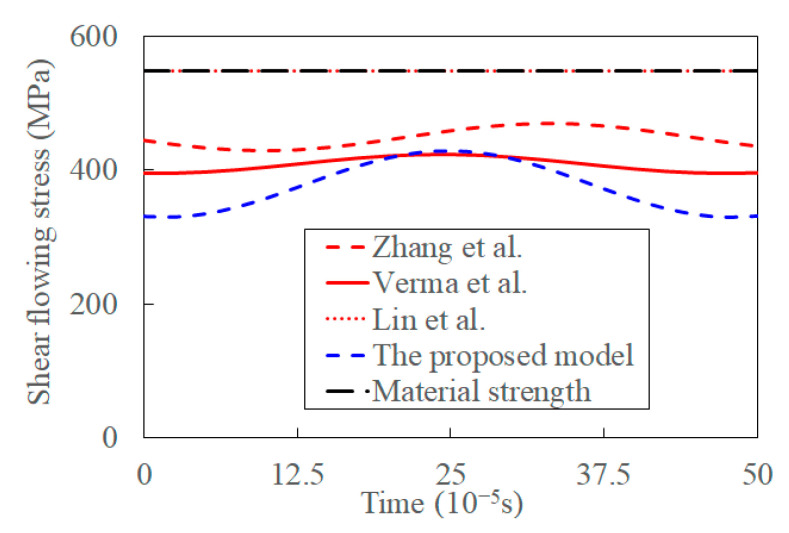
The shear is flowing stress in ultrasonic vibration-assisted orthogonal cutting.

Figure 14 shows the shear-flowing stress predicted by those models. The shear-flowing stress is constant and equal to the material strength, according to Ref. [37]. The predicted shear flowing stress decreases when the influences of shear angle, cutting thickness, and frictional reversal on the UVC process are considered in Ref. [36]. The shear is flowing stress, especially its fluctuations amplitude, reduces when a shear strain, temperature, and acoustic softening are considered in Ref. [39]. At the same time, the shear flowing stress decreases and its fluctuations increases in the proposed model where the coupled effect of the transient cutting process introduced by ultrasonic vibration is incorporated. The decreased shear flowing stress benefits chip formation, and the distance between chip segments decreases [6,65]. The influence of the cutting process, acoustic softening, and thermal softening on shear-flowing stress gradually increases.

**Figure 15:**
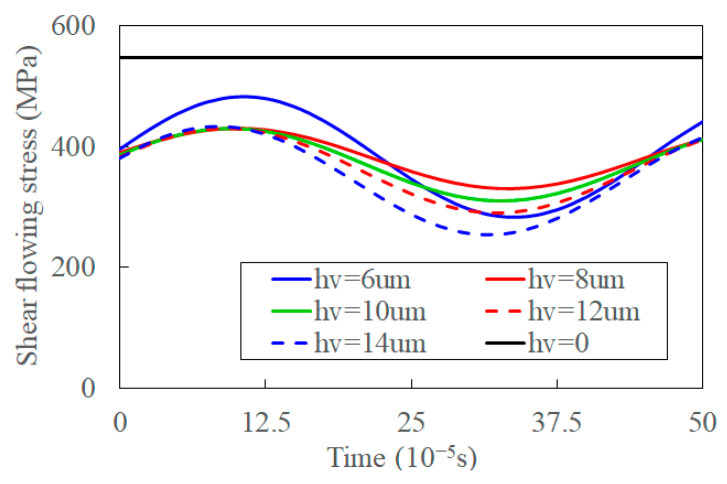
The shear is flowing stress with an increase of amplitude in UVC.

**Figure 16:**
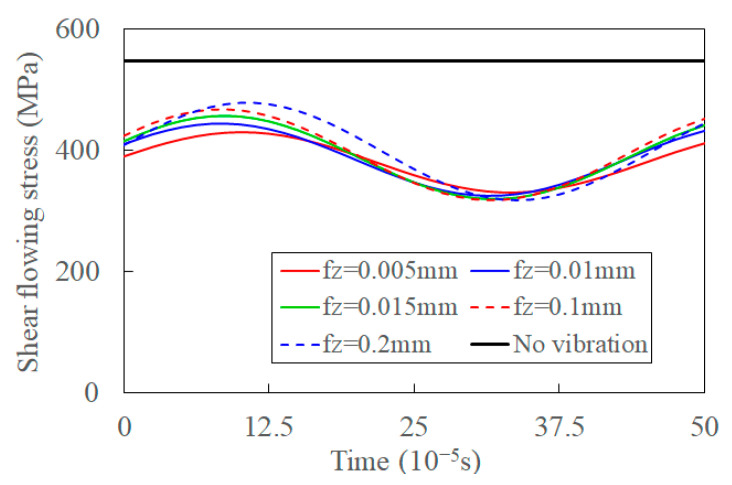
The shear flowing stress with increase of feed rate in UVC.

With the increase of amplitude, the transient and average shear flowing stresses decrease, and the fluctuation of it increases, as shown in Figure 15. The deformation in the shear band increases, and UVC formation process is more changeable by the increased synthetic cutting velocity with the increased vibration amplitude. This is verified by comparing experimental chip morphology under different amplitudes [65]. The averaged shear flowing stress decreases when the temperature of the shear band increases [16]. The decreased fluctuation of shear flowing stress implies that the increased amplitude promotes the separation of materials and fracture of chips by the shear angle in the UVC process. The shear flowing stress increases with un-deformed depth as the deformation area of the primary shear zone increases—the average deformation of the shear band and average thermal softening decrease [66]. Thus, the shear flowing stress, as well as its fluctuation, increase with the increase of feed rate, as shown in Figure 16.

#### 4.2.3. Transient Cutting Force

**Figure 17:**
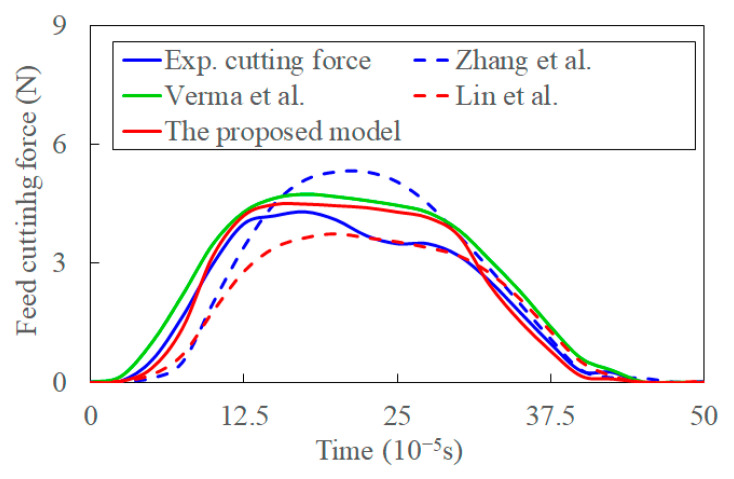
The predicted and experimental transient cutting force in the feed direction.

Figure 17 compares cutting forces predicted by the proposed model in this study, the models in Refs. [36,37,39], and experimental transient cutting force [40]. The transient cutting force increases with transient cutting thickness. The averaged error between the proposed model and experiments can be calculated with the input of experiments and prediction, and the error is about 10.2%. The transient cutting force is determined by mutually affected factors such as the transient cutting parameters, transient cutting process, deformation of the primary shear zone, and shear flowing stress. The cutting depth is minimal in the initial ultrasonic vibration cycle stage. The predicted force by the model in Ref. [36] is lower than the experimental value. This is caused by the inaccurate shear angle modeled by Lee and Shaffer’s slip-line solution, work-hardening, and size effects at the low cutting speed [67]. The size effect is caused by the ploughing and friction, increasing the experimental cutting force [23]. Moreover, the model only considers the transient cutting thickness and shear angle. When the cutting thickness reaches a critical value, the size effect decreases, and the percentage of cutting force contributed by the work hardening effect reduces. Simultaneously, the contribution of shear-flowing stress on cutting force increases [24]. The cutting force increases rapidly and is larger than the experimental value at a later stage of an ultrasonic vibration cycle. The transient cutting force calculated in Ref. [37] is based on the shear angle modeled by the maximum shear-flowing stress principle, and the coupling effect on shear-flowing stress is neglected. The shear angle contributes to the lower predictions of feed force to a certain extent. When the coupling effect of shear flowing stress, acoustic softening, and shear angle are considered in Ref. [39], the predicted transient cutting force is larger than the experimental one even in the initial stage of the ultrasonic vibration cycle. This means that the sights of transient physics-process affected by ultrasonic vibration are overestimated. The predictions of transient cutting force without consideration of transient cutting parameters introduced by ultrasonic vibration are larger than the experimental data. However, the hardening and size effects are not considered in the model. It implies that the increment of cutting force by the negligence of transient characters is larger than that caused by the size and hardening effects.

When the transient characters are considered in the proposed model, the predicted forces are much closer to experimental forces. While the error between the experimental and indicated cutting forces proliferates in the middle stage of a vibration cycle. This can be attributed to the instantaneous cutting thickness never reaching the given nominal uncut chip thickness in actual cutting [30]. Meanwhile, the resonant frequency may differ from 20 KHz in the UVC process, which may also contribute to the difference between predictions and experiments. The percentage of cutting force contributed by those transient characters is lower than that contributed by size and work-hardening effects before the cutting thickness reaches a critical value. With the coupled effect of those transient characters in UVC process, the impact of size and hardening effect can be included to a certain extent. The proposed model can incorporate the size and work-hardening effects in their coupling effect with shear-flowing stress. Those transient characters and their coupling effect advance the material removal in the UVC chip formation process.

### 4.3. Machining of Al 6063

**Figure 18:**
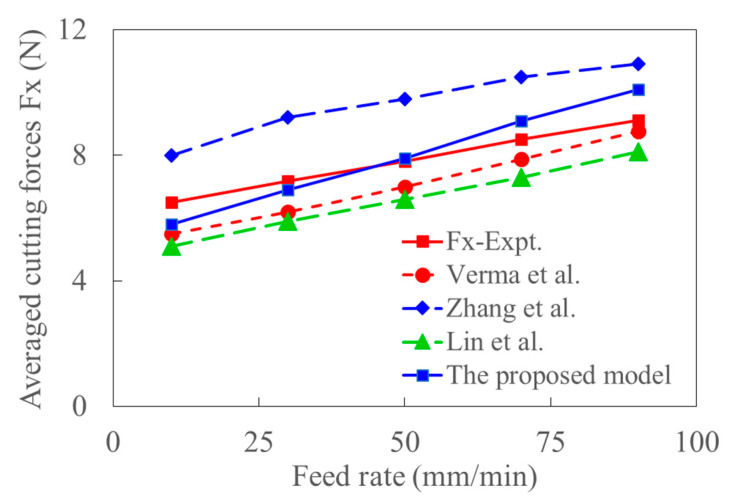
The comparison of predicted and experimental averaged cutting forces with the increase of feed rate (cutting depth: 0.6 mm, Amplitude: 12 um, RPM: 1875).

The average cutting force in milling of Al 6063 is predicted by the proposed model in this study and the analytical models in Refs. [36,37] are based on shear-flowing stress. The cutting force in the *x* direction is modeled based on the kinematics of the milling tool, and the corresponding experimental cutting force is obtained from Ref. [39]. Figure 18 shows that the average cutting force increases with feed rate. The sized effect introduced by plowing and friction resulted in a relatively more significant experimental value than the predicted ones when the coupling effect of shear-flowing stress is considered. The shear angle based on the maximum stress principle and friction angle cause the lower predictions when the shear flowing stress is constant. With the only consideration of transient cutting parameters and transient shear angle in Ref. [36], the predicted feed force is still more significant than that of the experiments. When the coupling effect of acoustic softening, constant shear angle, and flowing stress are considered in Ref. [39], the calculated force is smaller than the experimental one under a small feed rate. The deviation between predictions and experiments decreases with the increase of feed rate as the size effect gradually decreases. Similarly, when the transient characteristics introduced by ultrasonic vibration are considered in the proposed model, the predicted cutting force is closer to the experimental results. The trend of the deviation between the predicted and experimental cutting forces is determined by the changes in size effect and deformation with the increase of feed rate. The acoustic softening is of significance at low cutting speed. The predicted cutting force agrees well with the experimental data.

**Figure 19:**
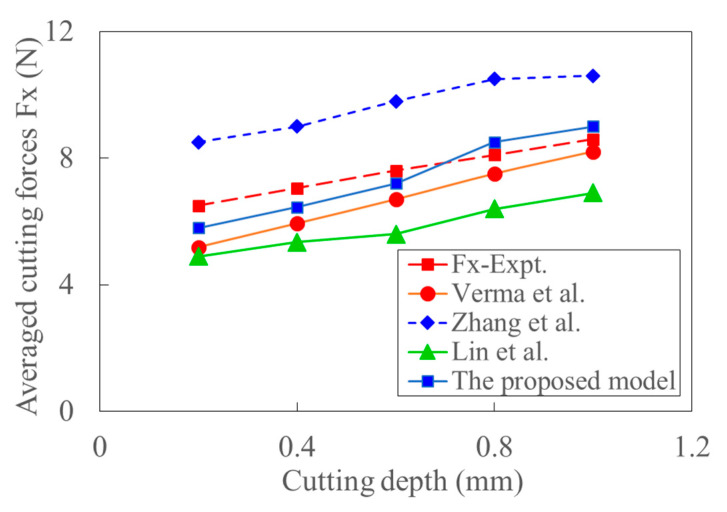
The comparison of predicted and experimental averaged cutting forces with the increased cutting depth (Feed rate: 50 mm/min, Amplitude: 12 um, RPM: 1875).

**Figure 20:**
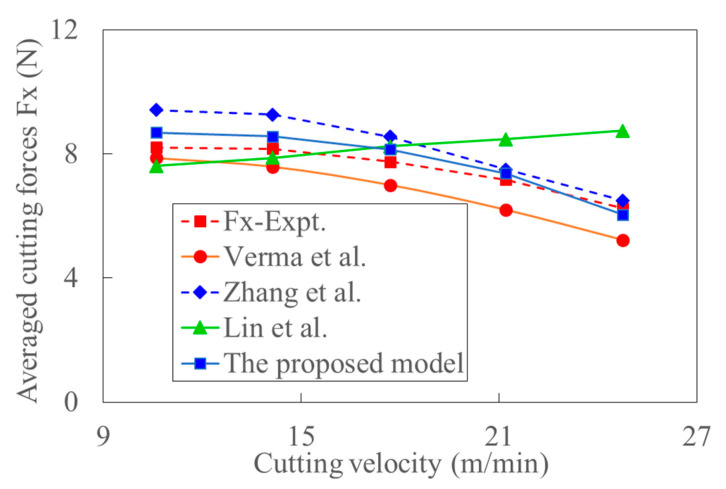
The comparison of predicted and experimental averaged cutting forces with increased cutting velocity (Feed rate: 50 mm/min, Cutting depth: 0.6 mm, Amplitude: 12 um).

**Figure 21:**
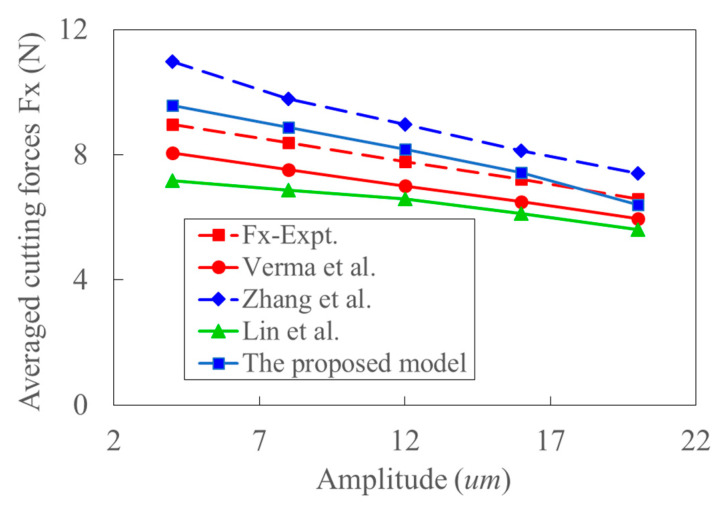
The comparison of predicted and experimental averaged cutting force with the increase of ultrasonic vibration amplitude (Feed rate: 50 mm/min, Cutting depth: 0.6 mm, RPM: 1875).

Figure 19 shows the variation of cutting force with cutting depth, the trend of which is almost the same as that in Figure 18. Figure 20 shows the variation of cutting force with cutting velocity. It can be seen that the cutting forces decrease with the increase of cutting velocity. The effect of deformation and temperature on shear stress is neglected in Ref. [37]. The corresponding influence would be enlarged with the increase of cutting velocity. The work-hardening effect decreases, and thus, the experimental cutting force decreases with the cutting velocity. This might be the reason why the predicted cutting force by the model in Ref. [37] gradually increases from more minor to larger than the experimental data. Since the effect of strain, strain rate, and temperature on shear stress increase with cutting velocity, the negligence of those effects would lead to the larger deviation between the model’s predicted and experimental cutting forces in Ref. [39]. Since the acoustic softening effect weakens with the increase of cutting velocity, the predicted cutting force by the model in Ref. [36] is close to the experimental cutting force, especially at high cutting velocity. With the increase of cutting velocity, the reduction of cutting force caused by thermal softening and the size effect increases. This is the primary source of the decreased trend of cutting force with cutting speed in the UVC process as shown in Figure 20. Figure 21 shows that the cutting force also decreases with the increase of ultrasonic amplitude in UVC. The average deviation between the experiment and prediction calculated by the proposed model is about 11%. The resultant cutting velocities increase with ultrasonic vibration amplitude. At the same time, the cutting force decreases with the increment of tool velocity and thermal softening. Relative high resultant cutting velocity and large ultrasonic vibration amplitude would result in a much apparent thermal softening effect, more idle contact time, and larger shear flowing stress. And the decrease of dislocation extension depth induced by ultrasonic vibration might also contribute to this physical phenomenon [68]. This is consistent with conclusion that the increased ultrasonic power enhances the acoustic softening effect and reduces the cutting force [39]. The size effect can be considered by coupling the analysis of transient characters and shear flowing stress.

## 5. Conclusions

The research presented a full transient cutting force model and provided insight and understanding of the physical phenomenon in UVC. This gives theoretical support for the optimization of cutting processing parameters in ultrasonic vibration cutting and breaks through cutting speed limitations in UVC. The effectiveness of the proposed model was verified by comparison with experimental results and analytical models available in the literature. The main conclusions can be summarized as follows.

(i) The hardening and thermal-softening effects are enhanced, and the acoustic softening effect weakens with cutting speed, which makes the titanium material in the cutting zone challenging to fracture. The reduction rate of cutting force induced by ultrasonic vibration decreases with cutting speed. The intermitted cutting process and enhanced thermal softening effect is the main reason for the initial decrease and following increase of cutting force when the cutting speed is beyond the critical speed.
(ii) The significant order of factors influencing the shear angle in UVC is thermal softening > shear flowing stress > acoustic softening >transient cutting parameters. For the shear-flowing stress, the significance order changes to be thermal soften > acoustic soften > the transient cutting parameters. The contribution of ultrasonic vibration-induced alternation of transient cutting characteristics, including cutting parameters, shear angle, and shear stress, to the decrease of cutting force, is more significant compared with that caused by size and hardening effect. With the consideration of the coupling effect of transient cutting characteristics, the proposed model can take the size effect and work hardening effect on cutting force into account.
(iii) The shear-flowing stress and its fluctuation increase with feed rate. With the rise in the strain, strain rate, and temperature in the shear zone, the average acoustic softening decreases, and the thermal softening effect enhances. The impact of acoustic softening on cutting force weakens while the thermal effect and size effect increase with the increment of cutting velocity. Acoustic softening is found to dominate at low cutting speed.
(iv) The introduction of ultrasonic vibration results in more significant increases in shear angle and lower material stress. Both the cutting force and the shear angle fluctuations decrease, and shear stress fluctuation increases when the coupling effect of more factors is taken into account.

## Figures and Tables

**Table 1:** Thermal properties of work-piece [39,60,61].

Workpiece	Density (kg/m^3^)	Thermal Conductivity (W/m.K)	Specific Heat Capacity (J/Kg.k)	Thermal Diffusivity (m^2^/s)
Al 6063	2700	200	900	82 × 10^−6^
Ti6Al4V	4430	6.91	520	3 × 10^−6^
AISI 1045	7860	48.3	473	13 × 10^−6^

**Table 2:** Testing results for ultrasonic vibration apparatus.

	Apparatus with Cutter	Apparatus without Cutter
Frequency (Hz)	20,571	20,698
Impedance (Ω)	10.68	10.55

**Table 3:** Cutting parameters in UVC experiments.

Spindle Speed (rpm)	Cutting Velocity (m/min)	Cutting Depth (mm)	Feed Rate per Rotation fz (um)	Amplitude (um)
112, 160, 350,500	41.5, 59.3, 129.7, 185.26	0.3	10	14.675

**Table 4:** Johnson-Cook parameters of workpieces [39,60,63].

Workpiece	A	B	m	n	c	${\dot{\varepsilon }}_{0}$	T_m_
Al 6063	324	114	0.002	0.42	1.34	1	910 K
AISI 1045	553.1	600	0.0134	0.234	1	1	1733 K
Ti6Al4V	783	497	0.028	0.28	1	1 × 10^−5^	1880 K

**Table 5:** Composition of Titanium alloy workpiece.

Element	Al K	Ti K	V K	Cu K	Fe K	Tatal
Weight%	4.17	91.22	0.18	0.88	−0.01	100.00
Atomic%	6.71	89.23	3.42	0.65	0.00	–

**Table 6:** Acoustic softening constants of workpiece and heat convection.

Workpiece	Acoustic Softening Constant 1	Acoustic Softening Constant 2	Thermal Softening Coefficient	Fraction of Plastic Work Converted to Heat	Factor that the Work Done Outside the Thin Shear Zone	Taylor-Quinney Coefficient
Al 6063 [39]	2.16 × 10^−8^	1	0.00166	0.9	–	–
AISI 1045	3.03 × 10^−9^	1	0.001 [64]	–	0.7 [60]	–
Ti6Al4V	–	–	–	–	–	0.85 [60]

## Data Availability

Not applicable.

## Appendix A

As the cutting forces are measured based on the coordinate system without ultrasonic vibration, the forces should be normalized to this coordinate system. Figure 4 shows the relationship between coordinates in synthetic cutting speed and cutting speed in traditional machining. The relationship between two coordinate systems can be established by the relationship between the synthetic cutting speed and the cutting velocity in conventional machining. The relationship of feed and tangential cutting forces in the two related coordinate systems in ultrasonic vibration-assisted cutting is presented in Figure 5.

The resultant force in the synthetic cutting speed coordinate systems is expressed as.

(A1) $$F_{c,v}=\sqrt{F_{t,t}{}^{2}+F_{f,t}{}^{2}}$$

The angle between the resultant force in synthetic cutting speed coordinate systems and that in coordinates systems of traditional cutting speed is the angle $\theta_{1}$. The tangential and feed cutting force in coordinates of traditional cutting speed, which is equal to the measuring coordinates in orthogonal cutting are expressed as.

(A2) $$F_{tc}=F_{c,v}\cos(\beta_{v}-\alpha_{v}+\theta_{1})$$

(A3) $$F_{fc}=F_{c,v}\sin(\beta_{v}-\alpha_{v}+\theta_{1})$$

(A4) $$F_{c}=\sqrt{F_{t,c}{}^{2}+F_{f,c}{}^{2}}$$

According to the developed tool angle conversion based on back rake-side rake and the oblique cutting model in Ref. [56], the theoretical cutting forces in x, y, z directions are developed as

(A5) $$F_{z}=F_{c}(\cos\theta_{i}\cos(\theta_{n}+\theta_{1})\cos i+\sin\theta_{i}\sin i)$$

(A6) $$F_{x}=F_{c}\cos\theta_{i}\sin(\theta_{n}+\theta_{1})$$

(A7) $$F_{y}=F_{c}(\sin\theta_{i}\cos i-\cos\theta_{i}\cos(\theta_{n}+\theta_{1})\sin i)$$

## Appendix B

According to the formulations for cutting force in oblique cutting, the force components in the directions of cutting speed, feed rate and cutting depth are presented as.

(A8) $$F_{tc}=\tau bh\frac{\left(\cos\theta_{n}+\tan\theta_{i}\tan i\right)}{[\cos(\theta_{n}+\phi_{n})\cos\phi_{i}+\tan\theta_{i}\sin\phi_{i}]\sin\phi_{n}}$$

(A9) $$F_{fc}=\tau bh\frac{\sin\theta_{n}}{[\cos(\theta_{n}+\phi_{n})\cos\phi_{i}+\tan\theta_{i}\sin\phi_{i}]\sin\phi_{n}\cos i}$$

(A10) $$F_{rc}=\tau bh\frac{\left(\tan\theta_{i}-\cos\theta_{n}\tan i\right)}{[\cos(\theta_{n}+\phi_{n})\cos\phi_{i}+\tan\theta_{i}\sin\phi_{i}]\sin\phi_{n}}$$

where τ is the shear flowing stress in metal cutting, *b* is chip width, *h* is un-deformed chip depth.

Based on the maximum shear flowing stress principle, the two formulations are presented as.

(A11) $$\cos(\phi_{n}+\theta_{n})=\frac{\tan\theta_{i}}{\tan\phi_{i}}$$

The calculation procedure is presented clearly in Ref. [56]. In the experiments of machining AISI 1045, the rake angle is 0°, the nominal clearance angle is 7°. The averaged feed force (Axial direction) is 1.99 N, the cutting force in the cutting depth direction (radial direction) is 3.16 N. Then, the stress in ultrasonic vibration cutting is calculated. The acoustic softening constant can be further determined based on the analysis in Section 2.3.
